# Effect of Fat to Lean Meat Ratios on the Formation of Volatile Compounds in Mutton Shashliks

**DOI:** 10.3390/foods12101929

**Published:** 2023-05-09

**Authors:** Mingcheng Zhang, Mingyang Li, Fangfang Bai, Wensheng Yao, Litang You, Dengyong Liu

**Affiliations:** 1 College of Food Science and Technology, Bohai University, Jinzhou 121013, China; zhangmc_1982@163.com (M.Z.); lmy1128712@163.com (M.L.);; 2 Anshan Jiuguhe Food Co., Ltd., Anshan 114100, China; 3 Jiangsu Collaborative Innovation Center of Meat Production and Processing, Quality and Safety Control, Nanjing 210095, China

**Keywords:** oral processing, meat bolus, electronic nose, exhaled breath

## Abstract

This study aimed to investigate the release of volatile compounds in mutton shashliks (named as F_x_L_y_, x-fat cubes: 0-4; y-lean cubes: 4-0) with different fat–lean ratios before and during consumption, respectively. In total, 67 volatile compounds were identified in shashliks using gas chromatography/mass spectrometry. Aldehyde, alcohol, and ketone were the major volatile substances, accounting for more than 75% of the total volatile compounds. There were significant differences in the volatile compounds of mutton shashliks with different fat–lean ratios. With the increase of the fat content, the types and content of volatile substances released also increase. However, when the percentage of fat exceeded 50%, the number of furans and pyrazine, which were characteristic of the volatile compounds of roasted meat, was decreased. The release of volatiles during the consumption of mutton shashliks was measured using the exhaled breath test and the results showed that adding an appropriate amount of fat (<50%) helps to enrich the volatile compound components in the mouth. However, shashliks with higher fat–lean ratios (>2:2) shorten the mastication duration and weaken the breakdown of bolus particles in the consumption process, which is not conducive to the release potential of volatile substances. Therefore, setting the fat to lean ratio to 2:2 is the best choice for making mutton shashliks, as it (F_2_L_2_) can provide rich flavor substances for mutton shashliks before and during consumption.

## 1. Introduction

Mutton shashlik is a popular food in China because of its unique flavor [1]. These unique flavors are related to the volatile compounds released by mutton during roasting. Changes that occur in meat roasting include pyrolysis of peptides and amino acids, degradation of sugar and ribonucleotides, Maillard’s and Strecker’s reactions, lipid oxidation, as well as the degradation of thiamine and fats, and these reactions promote the production of volatile compounds [2,3]. In general, the type and content of these volatile compounds are often key factors affecting consumers’ preference for roasted meat aroma.

There are both lean and fat cubes in mutton shashlik and the assignment of the fat–lean ratio is considered to be an important factor affecting the odor intensity of meat products [4,5,6]. This is because meat flavor is created by compounds that are derived from either lean or fat tissues. There is a difference in the contribution of fat and lean meat to the flavor of roast meat, which can be divided into two categories: the common meat flavor to all species of animals and the specific flavor of animals [7,8]. Sulfur-containing amino acids in lean meat, such as cystine and cysteine, contribute to the volatile compounds of roasted meat; however, it is generally thought that these contributions mainly involve the common meat flavor [9]. For example, volatile compounds isolated from the lean mutton as well as the physical data obtained for compounds completely characterized are similar to those obtained from lean beef and pork, but do not reflect the unique flavor of mutton [8]. However, fat acts as a substrate for the formation of odor-active volatiles from lipid oxidation, and its hydrocarbons, alcohols, ketones, and aldehydes are critical for producing the characteristic flavor of roasted meat [6]. Due to the significant difference in volatile substances produced by fat and lean meat, for the mutton shashlik, the different proportions of fat and lean meat added may have a significant impact on the flavor of lamb skewers. However, in daily life, the proportion of fat and lean meat used for mutton shashlik is uncertain and arbitrary. Therefore, it is necessary to clarify the proportion of fat and lean meat added to mutton shashlik through research, in order to promote the release of volatile flavor compounds and enhance consumers’ perception of aroma.

Currently, the analysis of volatile flavor compounds in food is mostly focused on the food before intake. People’s perception of these volatile substances is carried out through ortho-nasal perception. In fact, humans’ perception of aroma includes not only ortho-nasal perception, but also retro-nasal perception [10]. Retro-nasal perception occurs in humans while eating and the volatile compounds transfer from food to saliva, then pass through the oro-nasal cavity and finally reach the olfactory receptor, forming the retro-nasal perception [11]. Currently, various studies on the ortho-nasal aroma of roasted mutton have been conducted; however, studies on the retro-nasal perception of roasted mutton/mutton were insufficient. In fact, retro-nasal perception plays a crucial role in human perceptions of different food aromas because the flavor characteristics of food consumption are primarily detected in the retro-nasal cavity [12]. Therefore, a comprehensive understanding of the release of volatile compounds from mutton shashliks should be studied from two aspects: pre-consumption and during consumption.

The aim of this work was to investigate the release of volatile compounds from mutton shashliks with different fat–lean meat ratios. Therefore, in this study, in addition to analyzing the volatile flavor compounds that can be released into the environment after roasting, the release of volatile compounds from mutton shashliks during oral processing is also emphasized. At the same time, by measuring the changes in the oral processing parameters of mutton shashliks caused by the different proportions of fat and lean meat, the reasons for the differences in volatile substances released in the oral cavity were deeply analyzed. This study could provide a comprehensive and clear understanding of the changes in the aroma release of mutton shashliks with different fat to lean ratios before and during consumption, providing a theoretical basis for improving the flavor of mutton shashliks.

## 2. Materials and Methods

### 2.1. Mutton Shashlik Preparation

**Figure 1:**
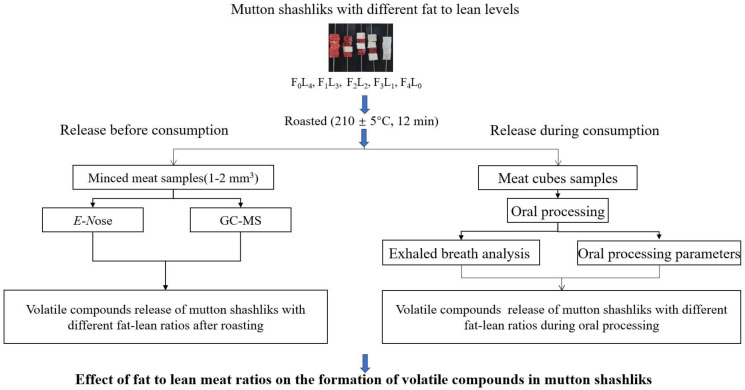
Schematic diagram showing the experimental design.

The hindquarter meat and the belly fat from 12-month-old Sunit sheep were purchased from Yang-yang Animal Husbandry Co., Ltd. (Inner Mongolia Autonomous Region, China), and was transported to the laboratory through the cold chain (−18 °C) as the raw material to produce mutton shashliks. The feeding conditions of these sheep before slaughter were the same, and the slaughtering was performed according to the China standard protocols of GB 2707–2016. Hindquarter meat (20.0 kg, boneless mutton, 6–7 kg per batch) and the belly fat (16.0 kg, 5–6 kg per batch) were randomly selected from 20 male sheep carcasses (31.5 ± 1.5 kg) and randomly assigned to each batch of mutton shashliks. The fat and lean meat were all cut into 10 × 10 × 10 mm^3^ cubes, which were skewered on an iron skewer (20 cm length) with different fat–lean ratios. The ratio of fat and lean meat in different samples were set as shown in Figure 1: 4 lean cubes (F_0_L_4_), 1 fat cube, and 3 lean cubes (F_1_L_3_); 2 fat cubes and 2 lean cubes (F_2_L_2_); 3 fat cubes and 1 lean cube (F_3_L_1_); and 4 pieces of fat (F_4_L_0_). However, mutton shashliks with all fat do not exist in real-life consumer contexts, and this sample was only used as a control. In total, 606 mutton shashliks were prepared for each batch (150 for F_0_L_4_, F_1_L_3_, F_2_L_2_, and F_3_L_1_, and 6 for F_4_L_0_) for each experiment (15 for *E*-*N*ose, 15 for GC-MS, and 576 for oral processing parameter determination and exhaled breath analysis). The raw shashliks were frozen (−18 °C) and taken out, thawed, and roasted before each test (same processing conditions). The shashliks were roasted in an electric oven 10 cm from the heating tub (NB-HM3810) at 210 ± 5 °C for 12 min while turning over the shashliks every 30 s. For all the roasted mutton shashliks, the moisture content, crude fat, and crude protein were determined based on the method as described by the AOAC Method 930.15 (2000), the AOAC Method 920.39 (2000), and the AOAC Method 954.01 (2000) [13].

All four roasted meat (fat and/or lean meat) cubes were separated from the iron skewers and cut into minced meat (approximately 1–2 mm^3^). The minced meat was homogeneously mixed and put into a cone of material, which was then flattened as evenly as possible. The material was then divided into quarters, whereupon two opposite quarters were discarded. The remaining two quarters were recombined, and we repeated the above operation until the weight of the remaining sample met the test requirements. These minced meat samples were used for the subsequent *E*-*N*ose analysis and GC-MS testing.

### 2.2. E-Nose Analysis

The *E*-*N*ose device PEN3 system (Win Muster Air-sense Analytic Inc., provided by AIRSENSE Company, Schwerin, Germany) consisted of 10 sensor probes, including W1C (aromatic compounds), W5S (nitrogen oxides), W3C (ammonia and aromatic compounds), W6S (hydrogen), W5C (alkanes and aromatics), W1S (methane, broad range of compounds), W1W (sulfur compounds, terpenes), W2S (alcohols, aldehydes, and ketones), W2W (aromatics and organic sulfur compounds), and W3S (long-chain alkanes) (we have presented this information in the manuscript and submitted it through the Supplementary Materials). Our analysis of the information from the mutton shashliks using an electronic nose was based on Gong’s method [14] with appropriate modifications. All determinations were conducted in triplicate and analyzed using principal component analysis (PCA).

### 2.3. Volatile Compounds

#### 2.3.1. HS-SPME Analysis of Mutton Shashliks before Consumption

The determination of volatile compounds was carried out according to Vasta’s method [15] with appropriate modifications. GC-MS analysis was operated using an Agilent 7890 A gas chromatograph (Agilent Technologies, Santa Clara, CA, USA) coupled to an Agilent 5973 C mass spectrometer. The following conditions were used: For the SPME analysis, desorption was at 280 °C for 4 min in splitless mode and MS was detected with no solvent delay; the ion source and quadrupole were set at 230 °C and 150 °C, respectively. The mass spectra were obtained in electron ionization mode (70 eV), and the range of mass spectrum scanning was 30–550 *m*/*z*.

#### 2.3.2. Identification of Volatile Compounds

All volatile compounds were tentatively identified after comprehensive evaluation by comparing the results with the NIST 14 spectrum library and standard compound linear retention index (LRI) [16]. The semi-quantitative method with cyclohexanone as an internal standard was employed to determine the volatile compounds content. The peak area of volatile flavor substances was compared with that of the internal standard, and the absolute concentration of each substance was calculated. The LRI was calculated based on the retention of a homologous series of n-alkanes (C_7_–C_40_).

#### 2.3.3. Subjects

In total, 36 volunteers with a good dental status were recruited to participate in the study, and 12 subjects (5 females and 7 males) were selected for their similar oral physiological parameters (mastication duration, number of mastication cycles, and saliva incorporation) and reproducibility. Each subject participated in two individual sessions of oral processing measurements and exhaled breath collection. The subjects were not allowed to smoke, eat, or drink for one hour before the test session. Prior to the study, each subject was informed of the project and the purpose of the experiment to obtain their signed consent.

#### 2.3.4. Oral Processing Parameters

The oral processing experiment was carried out in the sensory evaluation room, and the mutton shashliks were prepared in an independent preparation room. Each subject was given one freshly prepared shashlik for each oral processing session. Because there are almost no mutton shashliks made entirely of fat meat, the oral processing experiment and subsequent exhaled breath test for F_4_L_0_ was not performed in this study.

The subjects were asked to eat all four meat cubes from the same shashliks (60 ± 1 °C, meat cubes weighed, WW_meat_, about 8.5 g) in one bite and chew them in a natural way. When the subjects thought they were ready to swallow, they raised their hands, which meant that the oral processing experiment was over. The whole oral processing was recorded by the camera opposite the subjects. From this recording, the total mastication duration and number of mastication cycles (N) were determined. Total mastication duration refers to the time from the beginning of bite to the swallowing point. The number of mastication cycles was defined after a complete opening–occlusion sequence.

#### 2.3.5. Bolus Collection and Saliva Incorporation

Bolus collection and saliva incorporation were performed according to Pematilleke’s method [17] with some modifications. After they raised their hands, the subjects were asked to spit out the boluses and saliva into a pre-weighed plastic container, which was weighed again just after collection to calculate the wet weight of the bolus (WW_bolus_, g) and the saliva incorporation (W_saliva_, g). During the study, we also considered possible meat juice loss, partial swallows, and particles retained in the mouth after spitting. Therefore, the corrected mass of the bolus (WWt_bolus_corrected_, g) was calculated according to the following Equation (1):

**WWt**_bolus_corrected_ = WWt_bolus_ + (DWt_meat_ − DWt_bolus_) (1)

DWt_meat_ represents the dry matter of meat cubes, and DWt_bolus_ represents the dry matter in boluses. The sample was placed in a dry aluminum tray (90 mm in diameter), dried in a drying oven (105 °C) for 2 h (Shanghai Yiheng Instrument Co., Ltd., Shanghai, China), and finally transferred to a dryer to cool to room temperature before weighing. The above procedure was repeated until the difference in weight before and after drying was less than 0.005 g. The moisture content determination was carried out by three independent replicates [18].

The mass of saliva incorporation (WW_Salivary incorporation_, g) was expressed as the following Equation (2):

WW_Salivary incorporation_ = WWt_bolus_corrected_ − WW_meat_ (2)

WW_meat_ represents the wet weight of meat cubes in a whole mutton shashlik.

#### 2.3.6. Sieve Analysis of Meat Bolus

The meat bolus particle size distribution was measured using Pematilleke’s wet sieving method [17] with some modifications. The collected meat bolus was transferred to a beaker containing 100 mL distilled water and was stirred for 2 min with a magnetic stirrer at 200 rpm (Shanghai Tianping Instrument Co., Ltd., Shanghai, China). After suspending in water, each bolus was processed through a series of 5 sieves (0.6, 1.6, 3.0, 4.0, and 5.0 mm; Shaoxing Huafeng Instrument Co., Ltd., Shaoxing, China). Each sieve was eventually oven dried at 105 °C until a constant weight. The result was expressed as a percentage of the mass of the dry matter on each sieve and the combined weight of all the dry matter on the sieves.

#### 2.3.7. Image Analysis of Meat Particles

The 2.0 g sample was weighed from the collected food bolus and placed in a Petri dish (94 mm × 16 mm). Then, 15 mL ethanol (70%) was added to the Petri dish so that the bolus particles could be evenly dispersed. A CCD camera (Shenzhen Medway Vision Technology Co., Ltd., Shenzhen, China) was fixed on a bracket (ES400300, Dongguan LeTV Automation Technology Co., Ltd., Dongguan, China) and used to take pictures of the dispersed food particles. The radius, length, and width of the food particles were obtained by analyzing the image information using the Image-Pro 6.0 software (Media Cybernetics, Silver Spring, MD, USA).

### 2.4. Exhaled Breath Collection and Analysis

Before exhaled breath collection, we ensured the subjects fully understood the breath collection procedure and signed the informed consent. All breath collection experiments were conducted at a fixed time (9:30 a.m.–11:30 a.m.). The subjects were not allowed to smoke and consume alcohol a day before the test. Before breath collection, the subject needs to stay in the sensory evaluation room for at least 30 min. During the oral processing, the subjects were asked to keep their mouth tightly closed and to wear a nose clip to prevent them from breathing through their nose. When they needed to breathe, they could exhale the gas through into a 1 L Tedlar bag (Restek, Bellefonte, PA, USA). The Tedlar bag was made up of polyvinyl fluoride and a disposable mouthpiece and was inflated and deflated with N2 gas (99.99% purity) three times before the exhalation collection [19]. Then, the subject opens his/her mouth to inhale quickly and continues to chew and breathe until the end of oral processing. After exhalations collection, the valve of the Tedlar bag was immediately tightened to prevent leakage. Such exhalations contained mostly gas in the mouth; however, they also included some air from the respiratory system head space. We selected the Tedlar bag as the eventual sampling system because, according to the literature, they allow predictable results [20]. Simultaneously, the subject rinsed his/her mouth and rested for 15 min before the next collection. Each subject’s breath without food was collected for background correction according to their number of exhalations during oral processing. The subjects were always seated given that body position can change the composition of exhaled breath [21]. Additionally, exhaled breath samples should be stored at 4 °C and used within 12 h.

We penetrated the HS-SPME fiber (75 μm, carboxen/polydimethylsiloxane; Supelco, Bellefonte, PA, USA) into the Tedlar sampling bag and absorbed for 60 min at 40 °C, then we quickly transferred it to the GC-MS for analysis. The subsequent GC-MS analysis operation was the same as that in Section 2.3.1.

### 2.5. Statistical Analyses

The data were analyzed by the software IBM SPSS 19.0 (SPSS Statistical Software, Inc., Chicago, IL, USA), and the results were expressed through mean ± standard error (SE). Significant differences between means (*p* < 0.05) were determined by one-way analysis of variance (ANOVA) with Duncan’s multiple comparisons. Three batches of mutton shashliks with different fat–lean meat ratios (replicates) were prepared (cooking procedure was the same), and the experimental measurements were performed in triplicate for each batch. The GC-MS, oral processing parameters (mastication duration, number of mastication, saliva incorporation, and bolus practices analysis), and exhaled breath test for each treatment were analyzed using a mixed model. In this model, each replicate was included as a random effect, and different fat–lean ratios were included as fixed terms. To evaluate the potential of *E*-*N*ose to distinguish the odor profiles of roasted mutton shashliks with different fat–lean ratios, PCA was performed using the response values of *E*-*N*ose sensors. The data were normalized to make the variables comparable.

## 3. Results and Discussion

### 3.1. Moisture, Protein, and Fat Content

Table 1 showed the changes in the moisture, protein, and fat content of mutton shashliks with different fat–lean ratios after roasting. The moisture and protein contents of F_0_L_4_ were the highest among the five samples, which were 53.28 g/100 g and 41.17 g/100 g, respectively, but the fat content was the lowest, which was 3.54 g/100 g. When the mutton shashliks had more fat cubes, the fat content of F_4_L_0_ significantly increased, while the moisture and protein content gradually decreased until the whole mutton skewer was composed of fat pieces (a hypothetical situation), the moisture content and protein content of F_4_L_0_ decreased to 9.09 g/100 g and 1.01 g/100 g, respectively, while the fat content increased to 87.81 g/100 g.

### 3.2. E-Nose

**Figure 2:**
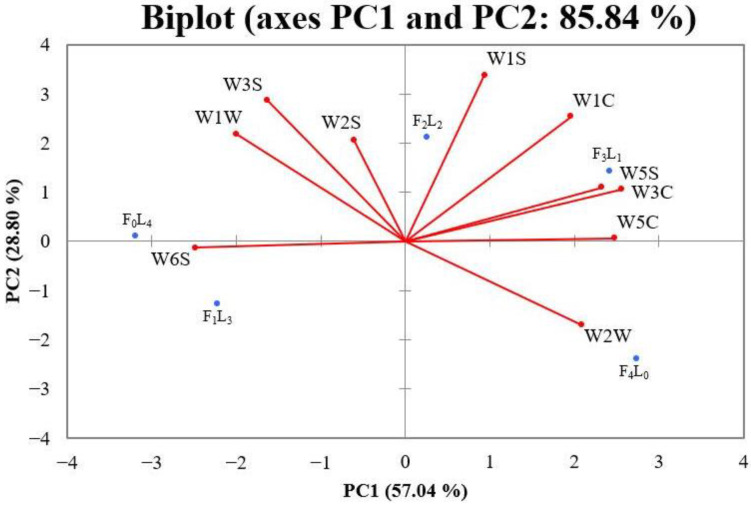
Typical response values of electronic nose (*E*-*N*ose) PCA results of roasted mutton shashliks with different fat–lean ratios. W1C: aromatic compounds, W5S: nitrogen oxides, W3C: ammonia and aromatic compounds, W6S: hydrogen, W5C: alkanes and aromatics, W1S: methane, broad range of compounds, W1W: sulfur compounds, terpenes, W2S: alcohols, aldehydes, and ketones, W2W: aromatics and organic sulfur compounds, and W3S: long-chain alkanes. F_0_L_4_ represented the mutton shashliks with 4 lean cubes; F_1_L_3_ represented the mutton shashliks with 1 fat cube and 3 lean cubes; F_2_L_2_ represented the mutton shashliks with 2 fats cubes and 2 lean cubes; F_3_L_1_ represented the mutton shashliks with 3 fat cubes and 1 lean cube; F_4_L_0_ represented the mutton shashliks with 4 fat cubes.

In this experiment, an electronic nose with ten sensors was used to obtain the odor information of different mutton shashliks. PCA is a statistical technique to reduce input data dimensions and is largely used for feature extraction [14]. It captures the relevant information in a set of input data and provides a lower dimension [22]. Principal component analysis can be used to analyze the electronic nose data, allowing an evaluation of the flavor attributes of shashliks with different fat–lean ratios [23]. As illustrated in Figure 2, the first principal component, PC1, was responsible for 57.04% of the total variation, while 27.80% of the total variance was explained by PC2. The total accumulative variance contribution rate of PC1 and PC2 was 84.84%, indicating that the first two principal components could explain the multi-index odor information of the vast majority of the samples. The figure shows that the distance between the four samples (F_4_L_0_, F_3_L_1_, F_2_L_2_, F_1_L_3_) was relatively far, indicating that different fat–lean ratios make the odor attributes of mutton shashliks vary considerably for the first principal component. F_0_L4 and F_1_L_3_ are located on the negative axis of PC1 and were mainly differentiated from the other samples by W6S. This suggested that the shashlik with a high proportion of lean meat (F_0_L_4_ and F_1_L_3_) contained more hydrogen compounds. When the fat proportion increased, the coordinate point of the samples transferred to the positive axis of PC1. The W2W, W5C, W3C, and W5S sensors provided stronger responses to the F_3_L_1_ and F_4_L_0_, which indicated that shashliks with a higher fat ratio (F_4_L_0_ and F_3_L_1_) would release more aromatic and organic sulfur compounds, short chain alkanes, alkane, and aromatics, ammonia and aromatic compounds, and nitrogen oxides. In the PC2, the W1W, W1S, W1C, W3S, and W2S sensors provided stronger responses to the F_2_L_2_ compared with F_1_L_3_ and F_4_L_0_, which suggested that the F_2_L_2_ contained more terpenes, inorganic sulfur compounds, methyl compounds, aromatic compounds, long-chain alkanes, alcohols, and carbonyls (aldehydes and ketones). Liu [10] also reported that the predominant odors of roasted lamb contained aldehydes, ketones, and alcohols by flash GC *E*-*N*ose. The electronic nose test shows that there are significant differences in the information of the volatile compounds of mutton shashliks with different fat–lean ratios.

### 3.3. Volatile Compounds of Mutton Shashliks with Different Fat–Lean Ratios

The volatile compounds of roasted shashliks with different fat–lean ratios are shown in Table 2. In total, sixty-seven volatile compounds were detected in all roasted muttons, including twenty-one aldehydes, sixteen alcohols, eight ketones, eight esters, fourteen hydrocarbons, three furans, and two pyrazines, which was similar to the profiles of volatile compounds in roasted mutton shashliks with Xu [6]. Among all the samples, the aldehyde, alcohol, and ketone contents were the highest, accounting for more than 75% of the total volatile substances (F_0_L_4_: 75.22%, F_1_L_3_: 87.96%, F_2_L_2_: 81.25%, F_3_L_1_: 83.08%, and F_4_L_0_: 82.6%). Among the three volatile compound groups, aldehydes have the highest content, mainly comprising glutaraldehyde, hexanal, heptanaldehyde, and octanal, followed by alcohols (1-octanol-3-ol was the main alcohol in mutton shashliks) and ketones, which is consistent with Liu’s results [24].

Twenty-four volatile compounds were detected in F_0_L_4_, including five alcohols, four ketones, one aldehyde, three esters, six hydrocarbons, four furans, one pyrazine, and two others. When fat was added to the shashlik, the volatile compound types of F_1_L_3_ significantly increased to thirty-six. Compared with sample F_0_L_4_, the shashlik with fat could release a higher abundance of volatile substances, including (E)-2-octal, (E)-2-Nonenal, trans, trans-2,4-nonadienal, Cuminaldehyde, (2E)-2-Decenal, 3-Cyclohexene-1-carboxaldehyde, (E)-2-Hexenol, 1-Nonanol, (E)-2-decen-1-ol, and 2-Cyclopenten-1-one,2-butyl-3-methyl-.

When the proportion of fat and lean meat was equal, the volatile compounds species in F_2_L_2_ sharply increased to fifty-five, including fifteen aldehydes, thirteen alcohols, five ketones, eight esters, ten hydrocarbons, one furan, and one pyrazine. Compared with F_0_L_4_ and F_1_L_3_, nine new odorants were detected in F_2_L_2_, including phenylacetaldehyde (honey sweetness), undecanal, lauraldehyde, 3-methyl-6-ethyl-5-octene-1-ol, 2,3-dimethyl-2-cyclopentene-1-one, gamma-caprolactone, ethyl octanoate, and ethyl 4-pentenoate. Alcohols were mainly derived from the oxidative degradation of lipids, while ketones mainly come from the oxidation of unsaturated fatty acids, which could give shashlik a pleasant fruity and flower-like aroma [6]. Therefore, the addition of fat meat plays a considerable role in enriching the aroma profile of mutton shashliks. After all, fat is the main source of mutton’s characteristic flavor, and you cannot get the unique flavor of roast lamb from lean meat alone [25]. Wasserman and Talley [26] found that only a few panels could correctly identify cooked lean mutton; however, the proportion of correct identifications for mutton improved upon adding 10% of fat. Therefore, the major species differences in the volatile compounds of heated mutton do not arise from fat rather than the lean extracts [27]. Fat acts as a substrate for the formation of odor-active volatiles from lipid oxidation, and its hydrocarbons, alcohols, ketones, and aldehydes are critical for producing the characteristic flavor of roasted meat [6]. Berry [28] reported that when comparing beef patties with 4% and 20% fat, the higher-fat samples had greater beef flavor scores. Therefore, the contribution of fat to the flavor of meat cannot be ignored.

Hexanal, heptanal, octanal, and nonanal were the major aldehyde volatile compounds in F_2_L_2_, and their contents were higher than that of F_1_L_3_, which may be because the increase in fat content was conducive to the enrichment of aldehydes in shashliks. Phospholipid in fat is rich in linoleic acid, arachidonic acid, oleic acid, eicosapentaenoic acid, and linolenic acid, among which the n-3 unsaturated fatty acids of linolenic acid and eicosapentaenoic acid can be oxidized to saturated and unsaturated aldehydes when heated [29]. Hexanal, octanal, and nonanal are roasted mutton’s main odor substances [24]. Due to the low threshold of aldehydes, they usually make a prominent contribution to the overall flavor of meat products [30]. Hexanal has a grass-like smell, while heptanal has a cheese- and fat-like aroma, and octanal elicits sweet and light fat odor qualities [31].

When the ratio of fat to lean meat reached 50%, the content of furans (5-Butyldihydro-2(3H)-furanone, 2-amylfuran, 2-butyl tetrahydrofuran, and 2,5-dimethyltetrahydrofuran) in mutton shashliks gradually decreased (*p* < 0.05). Furans mainly come from the degradation of thiamine in lean meat, providing a strong meat flavor, as well as a roasted flavor for roasted meat [32]. The changes in pyrazines were also similar to the furans. The odors of the alkylpyrazines are generally associated with pleasant roasted foods, e.g., chocolate, coffee, and roasted nuts [24]. This suggested that as the proportion of lean meat in mutton shashlik decreased some odorants that contribute to the roast flavor also decreased. When the fat–lean ratio exceeded 50%, the volatile substances content (especially aldehydes, alcohols, and ketones) further increased with the increase in fat–lean ratios. However, the types of volatile compounds in F_3_L_1_ and F_4_L_0_ did not increase more than F_2_L_2_. Thus, you do not have to add more than 50% fat to the shashliks.

### 3.4. Oral Processing

#### 3.4.1. Mastication Duration and Number 

**Figure 3:**
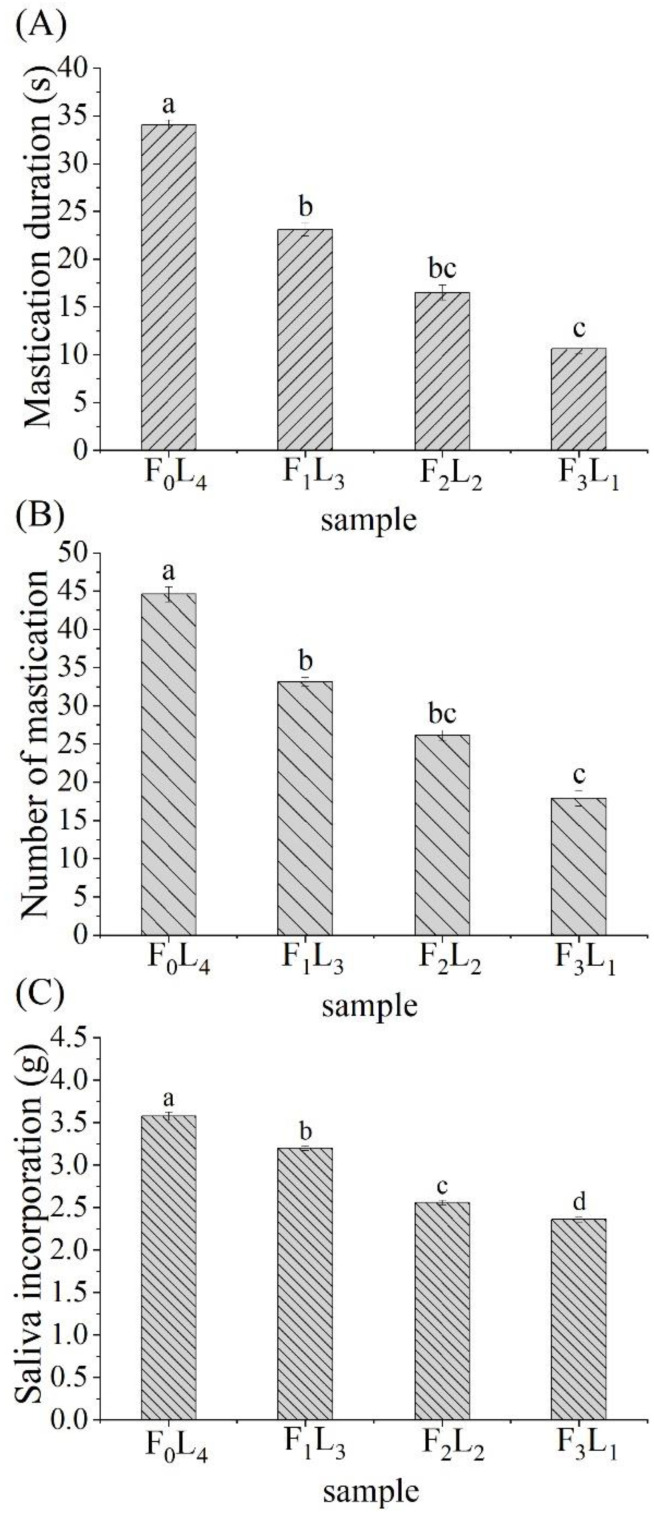
Oral processing parameters of shashliks with different fat–lean ratios, including mastication duration (**A**), masticatory number (**B**), and saliva incorporation (**C**). Different letters indicate that there is significant difference (*p* < 0.05). F_0_L_4_ represented the mutton shashliks with 4 lean cubes; F_1_L_3_ represented the mutton shashliks with 1 fat cube and 3 lean cubes; F_2_L_2_ represented the mutton shashliks with 2 fats cubes and 2 lean cubes; F_3_L_1_ represented the mutton shashliks with 3 fat cubes and 1 lean cube. Error bars represent S.E.M (standard error of mean).

The mastication of food is a series of motor sensory activities, where food ingested to the mouth is ground into small particles and transformed into a safe-to-swallow cohesive bolus [33]. This particle breakdown pattern and the bolus formation process can vary depending on changes in the initial food’s intrinsic characteristics. Figure 3 shows the changes in the oral parameters (including the mastication duration and masticatory number) of the subjects during the oral processing of roasted mutton shashlik with different fat–lean ratios. Figure 3A shows the mastication time required for F_0_L_4_ was 34.05 s, during which the subjects completed 44.58 mastication cycles. With the increase in the proportion of fat in shashlik, the mastication time gradually decreased. When the fat–lean ratio was 3:1 (the content of fat increased to 66.24 g/100 g), the mastication duration was significantly shortened (*p* < 0.05) to 10.61 s, and the masticatory number was also reduced (*p* < 0.05) to 7.92. The decreases in mastication time and number were related to the increase in fat content. Because fat meat is more tender than lean meat, the increase in fat would inevitably lead to the decrease in the overall hardness of the meat cubes, thereby weakening the resistance of food to teeth during chewing [33]. As the chewing behavior becomes easier, the time required for the meat cubes to be crushed to form a swallowable food bolus was shortened. Another possible explanation is the higher fat level helps to improve the lubricity of the food bolus, making it easier to achieve the safe swallowing threshold, thus shortening the oral processing (mastication) time from food intake to swallowing [34].

#### 3.4.2. Saliva Incorporation

Saliva flow relates to the texture and flavor perception of any food [35]. Therefore, understanding the critical role that saliva plays in bolus formation can help in elucidating the volatile compounds release mechanism of food. Figure 3C shows the amount of saliva incorporated into the food bolus after oral processing. The amount of saliva mixed into the food bolus of F_0_L_4_ was 3.58 g, which was gradually decreased (*p* < 0.05) with the increase in the ratio of fat to lean meat in the sample. Thus, when the ratio of fat to lean meat reached 3:1, the amount of saliva incorporated in the food bolus (F_3_L_1_) decreased to the lowest value, which was 2.36 g. The increases in fat content reduced the amount of saliva incorporation. Frank [36] reported that lower-fat meat had a greater capacity to absorb and hold saliva compared with high-fat samples, affecting bolus formation. Samples with a high lean meat content increased the mastication duration and masticatory number. It was previously found that when the masticatory number and mastication duration increased, the amount of saliva incorporation generally increased accordingly [17]. As a result, more saliva was required to agglomerate harder particles to make a safe-to-swallow and cohesive bolus. Moreover, another possible reason is that fat replaced saliva’s lubricating function, resulting in a decrease in its incorporation. Under normal conditions, a lubrication threshold is achieved through saliva incorporation [17]. However, fat cubes release oil as they are crushed during chewing, covering the bolus surface and improving its lubricity. Thus, even in the presence of a small amount of saliva, the pellet’s surface lubrication can still reach the threshold that triggers swallowing.

#### 3.4.3. Sieve Analysis of Meat Bolus

To explore the fragmentation of roast mutton shashlik with different fat–lean ratios after mastication, two different methods, namely wet screening and image analysis, were used to analyze the particle size distribution of the bolus particles. The wet sieve method is the most used to evaluate the particle size distribution of chewed food. It can intercept particles of different sizes using a sieve with different apertures, and then analyze the particle size distribution [37]. Based on the wet screen method, image analysis can further provide more intuitive and accurate information about particle distribution [38].

The particle mass percentage retained on the sieves with different aperture sizes (0.6–5 mm) is presented in Figure 3A. More than half of the particles were stained on the sieve with the largest aperture (5 mm), especially F_3_L_1_, which had an interception rate of 71.12%, which was significantly heavier than F_2_L_2_ (55.3%), F_1_L_3_ (52.47%), and F_0_L_4_ (40.52%). This result indicated that shashliks with a high fat level had larger particles after oral processing. However, particle breakdown was significantly higher in the samples with a large proportion of lean meat, and most of the fine particles were intercepted on the smaller-aperture sieves (0.6–4 mm). For example, the mass percentage of F_0_L_4_ particles retained on the sieves with 0.6 mm, 1.6 mm, and 3 mm aperture sizes was 1.79 times, 2.50 times, and 2.16 times that of F_3_L_1_, respectively. This might be because the meat cubes with a higher proportion of lean meat have been thoroughly chewed during oral processing, resulting in thinner muscle fibers that can more easily pass through the larger sieve apertures.

**Figure 4:**
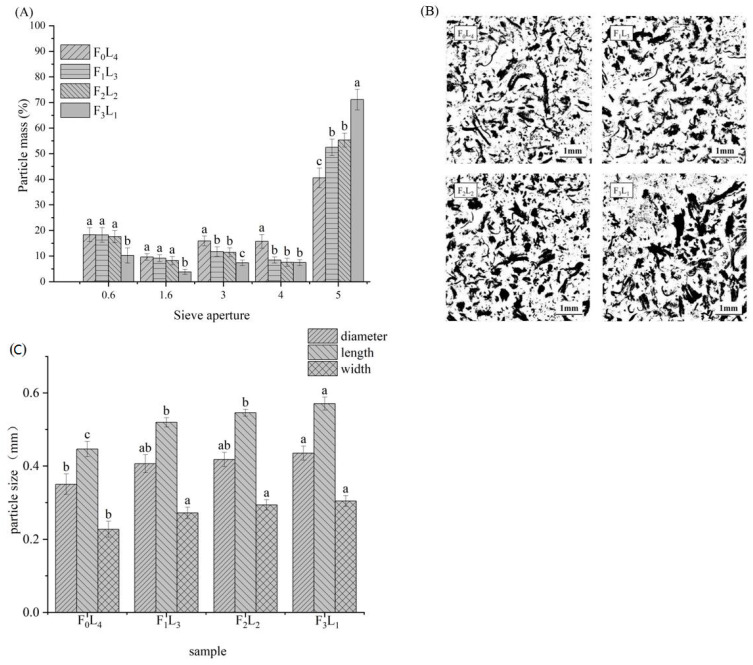
The mass of particle mass retained on each sieve after wet sieving of roasted mutton shashliks with four different fat–lean ratios after oral processing (sieve apertures range from 5–0.60 mm) (**A**). Optical microscopic images (×10 magnification) of particle masses collected in the receiving pan for four different fat–lean ratios of roasted mutton shashliks (**B**). The diameter, length, and width of the kebab particle masses of four different fat–lean ratios of roasted mutton shashliks after chewing were measured by software analysis (**C**). Different letters indicate that there is significant difference (*p* < 0.05). F_0_L_4_ represented the mutton shashliks with 4 lean cubes; F_1_L_3_ represented the mutton shashliks with 1 fat cube and 3 lean cubes; F_2_L_2_ represented the mutton shashliks with 2 fats cubes and 2 lean cubes; F_3_L_1_ represented the mutton shashliks with 3 fat cubes and 1 lean cube. Error bars represent S.E.M (standard error of mean).

Image analysis can provide more intuitive information about food particles, including the size, shape, etc. [39]. Figure 4B,C show the image analysis results for bolus particle size variation amongst the different shashliks. The image shows that the meat bolus particles after chewing are coarse and fibrous, which is consistent with Pematilleke’s results [17]. The variation in size of muscle fibers might have resulted from the intraoral manipulation of meat [40]. After chewing for the longest time, the muscle fibers of F_0_L_4_ were cut through bite force and eventually formed particles with the smallest diameter (0.35 mm), smallest length (0.447 mm), and smallest width (0.227 mm) of all the samples. With the increase in the proportion of fat in the sample, the diameter, length, and width of the particles began to increase continuously until the ratio of fat to lean meat was 3:1, and the particles of F_3_L_1_ were the largest observed in the sample, which was consistent with the results of the wet sieve method.

### 3.5. Exhaled Breath Analysis

Table 3 shows the volatile compounds in the subjects’ exhaled breaths after oral processing. In total, 21 volatile compounds were detected in all the samples, including 7 aldehydes, 2 alcohols, 2 esters, 7 hydrocarbons, and 1 pyrazine. At the initial stage of oral processing (bolus has not yet formed), meat cubes were broken down and food particles were highly dispersed. There was a larger exchange area between the meat particles and the gas phase in the mouth, which should be conducive to the release of volatile compounds into the oral cavity [41]. However, from the test results, the types of volatile compounds that release into the mouth were significantly less than before intake (56 species). We inferred that, on the one hand, the sampling temperature (about 60 °C) to determine the amount of volatile compounds in mutton shashliks was significantly higher than the sampling temperature for oral processing (close to the human oral temperature), so at relatively low temperatures, some volatile compounds may be difficult to release from meat to the mouth [42,43]. On the other hand, the release of retro-nasal volatile compounds is a complex physiological process. The decrease in volatile substances in exhalation may be related to changes in oral processing parameters, such as the oral processing duration, the amount of saliva incorporation, and the food’s morphological deformation during mastication, including crushing, grinding, disintegration, melting, and dilution by saliva [42,44]. Among these factors, the impact of saliva was evidenced, with an increase in saliva incorporation significantly decreasing the release of volatile compounds [42]. During food oral processing, the continuously secreted saliva coats surfaces and clusters particles, participates in the formation insoluble aggregates, and binds aromas.

The table shows that nine volatile compounds (five aldehydes, one alcohol, and one hydrocarbon) were detected in F_0_L_4_ exhalation, while no esters were detected. After adding fat, the volatile compounds in the F_1_L_3_ exhaled breath were more abundant than in F_0_L_4_. In addition to the increase in aldehydes, ethyl hexanoate, undecane, dodecane, hexadecane, and other substances began to be detected in the exhalation. When the fat ratio continued to increase, the types of volatile compounds in the F_2_L_2_ breath increased to 16, and alcohols, esters, hydrocarbons, and other compounds in the exhalation further increased compared with F_0_L_4_ and F_1_L_3_. At this time, the contents of hexal, octyal, heptal, nonaldehyde, ethyl caproate, cisanisole, and some alkanes in F_2_L_2_ were the highest of all the exhalation samples. This might be because F_2_L_2_ contains relatively more fat, more than three and four times higher than F_0_L_4_ and F_1_L_3_, respectively (Table 1). Mutton shashliks with higher fat content may release richer and more types of volatile compounds than those with lower fat content during oral processing. Moreover, differences in saliva incorporation caused by different samples during oral processing might influence the release of volatile compounds [45]. Because the subjects secreted more saliva when chewing F_0_L_4_ and F_1_L_3_ (Figure 4C), it may have hindered the release of volatile substances from the bolus into the oral cavity. Therefore, the types and amounts of volatile compounds released from F_0_L_4_ and F_1_L_3_ were less than those from F_2_L_2_.

Although increasing the fat content contributes to more volatile compounds being released into the oral cavity, the types and content of volatile compounds in F_3_L_1_ and F_2_L_2_ did not change significantly (*p* > 0.05) when the fat to lean ratio was further increased (Table 3). This result might be related to the effect of oral processing on the release mechanism of volatile compounds. When meat cubes were masticated, volatile compounds were released from food to saliva. Then, they were partitioned between saliva and the air phase in the oral cavity [16]. Because of the faster oral breakdown of F3L1, its degree of fragmentation was lower than that of F2L2, resulting in the formation of larger food pellets, which may weaken the release potential of volatile substances from meat. Additionally, in foods that contain fat, the retention and release of volatile compounds mainly depend on their solubility in the oil phase or hydrophobicity [45]. In some cases, lipophilic volatile organic compounds are typically more soluble in oil than water. Consequently, the release of volatile compounds in the mouth from low-fat foods was found to be higher than regular-fat foods [16,36]. Frank [36] found that fat at 37 °C also acts as a solvent or “flavor sink”, solubilizing lipophilic volatiles and impeding their in-mouth release. Therefore, even though the fat content of F_3_L_1_ increased by 25% compared with F_2_L_2_, the further increase in volatile compounds did not occur under the oral processing conditions.

## 4. Conclusions

This study showed that the difference in the ratios of fat and lean meat in mutton shashliks significantly affected the release of volatile compounds. The analysis of volatile substances released by mutton shashliks after roasting showed that the increase of fat content could enrich the type and content of volatile substances in mutton shashliks. F_2_L_2_ could provide more volatile substances for mutton shashliks than other samples. Analysis of the gases collected from the oral cavity showed that the increased ratios of fat cubes help to released more volatile substances from meat bolus into the oral cavity. However, when the fat addition level exceeded 50% (F_2_L_2_), the time required to chew the shashliks decreased and the bolus particles became larger, which inhibited the release of volatile compounds from the food bolus. In summary, mutton shashliks with the same proportion of fat and lean meat (F_2_L_2_) can release more volatile substances both before and during consumption.

## Figures and Tables

**Table 1:** Moisture, protein, and fat content of mutton shashliks with different fat–lean ratios (g/100 g).

	F_0_L_4_	F_1_L_3_	F_2_L_2_	F_3_L_1_	F_4_L_0_
Moisture	53.28 ± 2.81 ^a^	42.13 ± 2.31 ^b^	31.57 ± 2.19 ^c^	20.75 ± 1.31 ^d^	9.09 ± 1.24 ^e^
Protein	41.17 ± 1.71 ^a^	31.34 ± 1.56 ^b^	21.03 ± 1.97 ^c^	11.20 ±1.03 ^d^	1.01 ± 0.27 ^e^
Fat	3.54 ± 0.62 ^e^	25.26 ± 1.48 ^d^	46.21 ± 2.53 ^c^	66.24 ± 3.17 ^b^	87.81 ±4.28 ^a^

Different lowercase letters in the same row indicate that there is significant difference (*p* < 0.05). F_0_L_4_ represented the mutton shashliks with 4 lean cubes; F_1_L_3_ represented the mutton shashliks with 1 fat cube and 3 lean cubes; F_2_L_2_ represented the mutton shashliks with 2 fats cubes and 2 lean cubes; F_3_L_1_ represented the mutton shashliks with 3 fat cubes and 1 lean cube; F_4_L_0_ represented the mutton shashliks with 4 fat cubes.

**Table 2:** Volatile compounds in roasted mutton shashliks with different fat–lean ratios (μg/kg).

Volatile Compounds	LRI *		Identification ^+^	F_0_L_4_	F_1_L_3_	F_2_L_2_	F_3_L_1_	F_4_L_0_
	Literature	Calculated						
Hexanal	800	802	MS + LRI	150.47 ± 12.65 ^d^	649.30 ± 7.76 ^c^	687.64 ± 16.91 ^b^	697.77 ± 8.84 ^b^	713.10 ± 7.36 ^a^
Heptanal	900	904	MS + LRI	119.55 ± 6.65 ^e^	261.82 ± 8.97 ^d^	313.80 ± 7.80 ^c^	339.42 ± 6.73 ^b^	380.33 ± 3.79 ^a^
2-hexen-1-al	854	860	MS + LRI	ND	ND	ND	ND	111.84 ± 11.77
Benzaldehyde	959	963	MS + LRI	98.30 ± 9.89 ^d^	246.00 ± 12.37 ^c^	435.32 ± 10.63 ^b^	443.25 ± 19.60 ^b^	515.44 ± 11.54 ^a^
Octanal	1004	1005	MS + LRI	124.99 ± 2.93 ^e^	476.33 ± 3.74 ^d^	582.55 ± 8.12 ^c^	629.81 ± 3.20 ^b^	642.84 ± 6.07 ^a^
(E, E)-2,4-Heptadienal	1011	1012	MS + LRI	ND	ND	ND	ND	51.65 ± 9.70
Phenylacetaldehyde	1051	1053	MS + LRI	ND	ND	225.08 ± 15.24 ^c^	331.29 ± 12.58 ^b^	491.30 ± 12.38 ^a^
(E)-2-Octenal	1065	1067	MS + LRI	ND	144.96 ± 37.60 ^d^	291.13 ± 9.23 ^c^	346.03 ± 11.25 ^b^	335.25 ± 10.39 ^a^
Nonanal	1112	1114	MS + LRI	139.87 ± 18.15 ^d^	517.66 ± 12.06 ^c^	612.80 ± 17.22 ^b^	636.29 ± 15.17 ^a^	646.76 ± 10.22 ^a^
(E)-2-Nonenal	1163	1167	MS + LRI	ND	88.63 ± 12.87 ^e^	190.62 ± 10.13 ^b^	164.51 ± 16.77 ^c^	276.85 ± 12.91 ^a^
Decanal	1202	1205	MS + LRI	ND	110.67 ± 21.46 ^d^	215.67 ± 11.67 ^c^	269.43 ± 16.76 ^b^	281.88 ± 10.61 ^a^
(E, E)-2,4-Nonadienal	1212	1216	MS + LRI	ND	359.43 ± 20.46	ND	ND	ND
Cuminaldehyde	1238	1240	MS + LRI	ND	45.54 ± 28.03 ^d^	55.90 ± 3.61 ^c^	59.61 ± 4.78 ^b^	61.22 ± 6.58 ^a^
(2E)-2-Decenal	1265	1268	MS + LRI	ND	51.56 ± 20.95 ^c^	179.75 ± 27.12 ^b^	187.60 ± 14.19 ^b^	420.70 ± 17.54 ^a^
2,4-Decadienal	1295	1299	MS + LRI	ND	ND	ND	ND	28.83 ± 2.51
Undecanal	1305	1307	MS + LRI	ND	ND	142.98 ± 14.17 ^a^	85.58 ± 13.02 ^b^	72.49 ± 14.18 ^c^
Undecenal	1359	1360	MS + LRI	ND	ND	64.86 ± 8.36 ^b^	72.74 ± 8.98 ^b^	141.57 ± 11.02 ^a^
Dodecanal	1407	1410	MS + LRI	ND	ND	15.51 ± 7.62 ^b^	24.00 ± 4.25 ^b^	28.05 ± 6.94 ^a^
Pentadecanal	1715	1718	MS + LRI	ND	ND	ND	31.01 ± 11.82	34.10 ± 12.51
(E)-4-Nonenal	-	1435	MS	ND	ND	ND	ND	40.84 ± 12.88
3-Cyclohexene-1-carboxaldehyde	-	1490	MS	ND	53.88 ± 5.14 ^c^	70.71 ± 5.38 ^c^	102.03 ± 7.33 ^b^	132.64 ± 9.09 ^a^
(E)-2-Hexenol	849	853	MS + LRI	ND	32.23 ± 1.96 ^b^	55.32 ± 1.36 ^a^	66.49 ± 44.86 ^a^	ND
1-Hexanol	890	891	MS + LRI	34.25 ± 3.26 ^b^	56.46 ± 5.36 ^a^	40.36 ± 6.36 ^b^	ND	ND
1-Heptanol	969	973	MS + LRI	106.35 ± 5.17 ^d^	362.92 ± 8.46 ^c^	373.9 ± 16.01 ^c^	552.42 ± 10.63 ^b^	621.57 ± 16.02 ^a^
1-Octen-3-ol	981	984	MS + LRI	146.83 ± 8.8 ^b^	256.14 ± 8.49 ^a^	259.8 ± 17.27 ^a^	259.24 ± 11.87 ^a^	ND
1-Nonanol	1150	1152	MS + LRI	ND	62.33 ± 6.32 ^b^	82.69 ± 3.95 ^a^	88.33 ± 6.33 ^a^	ND
3-methyl-6-ethyl-5-octen-1-ol	-	1163	MS	ND	ND	133.48 ± 9.69	ND	ND
2-Propylcyclohexanol	-	1264	MS	ND	ND	ND	64.64 ± 2.34	107.55 ± 7.41
(E)-2-decen-1-ol	-	1250	MS	ND	45.69 ± 6.69 ^a^	70.93 ± 2.11 ^a^	52.36 ± 3.98 ^a^	38.21 ± 1.80 ^a^
Pentanol	762	760	MS + LRI	62.36 ± 6.56 ^d^	91.33 ± 7.32 ^c^	99.22 ± 6.96 ^c^	143.63 ± 9.65 ^b^	156.26 ± 8.65 ^a^
Cyclohexanol,3,5-dimethyl-	-	970	MS	ND	ND	ND	ND	272.69 ± 8.79
2-Methylcyclopentanone	846	850	MS + LRI	ND	ND	559.00 ± 17.1	ND	ND
2,3-Octandeione	986	990	MS + LRI	132.25 ± 5.23 ^d^	231.96 ± 7.36 ^c^	553.36 ± 25.33 ^a^	423.36 ± 7.32 ^b^	134.36 ± 6.00 ^d^
2-Nonanone	1089	1092	MS + LRI	ND	ND	ND	63.77 ± 7.66	171.66 ± 7.69
2-Decanone	1194	1198	MS + LRI	ND	ND	63.91 ± 0.90 ^c^	157.27 ± 80.66 ^b^	310.12 ± 17.26 ^a^
2-Undecanone	1296	1300	MS + LRI	ND	ND	ND	106.82 ± 7.99	217.96 ± 8.15
2-Tridecanone	1498	1500	MS + LRI	ND	ND	ND	30.19 ± 2.37	49.30 ± 4.73
2,3-dimethyl-2-cyclopenten-1-one	1035	1040	MS + LRI	ND	ND	694.55 ± 11.56 ^a^	119.71 ± 12.88 ^b^	63.41 ± 3.00 ^c^
2-Cyclopenten-1-one,2-butyl-3-methyl-	-	979	MS	ND	75.74 ± 8.90 ^b^	141.99 ± 9.41 ^a^	80.36 ± 6.33 ^b^	39.96 ± 6.26 ^c^
Ethyl hexanoate	998	1000	MS + LRI	134.20 ± 8.35 ^d^	199.84 ± 8.83 ^c^	226.51 ± 12.23 ^b^	379.16 ± 8.92 ^a^	350.14 ± 7.36 ^a^
γ-Caprolactone	1055	1058	MS + LRI	ND	ND	264.16 ± 10.24 ^a^	134.52 ± 7.88 ^b^	64.55 ± 5.90 ^c^
Ethyl heptanoate	1097	1100	MS + LRI	28.17 ± 2.98 ^d^	66.69 ± 9.25 ^c^	243.41 ± 9.68 ^b^	303.13 ± 10.10 ^a^	ND
Ethyl caprylate	1199	1200	MS + LRI	32.66 ± 5.20 ^d^	50.28 ± 8.20 ^c^	94.95 ± 8.34 ^b^	147.70 ± 9.35 ^a^	ND
Ethyl caprate	1397	1400	MS + LRI	ND	ND	40.05 ± 5.37 ^a^	32.26 ± 7.36 ^ab^	15.21 ± 5.23 ^c^
5-Butyldihydro-2(3H)-furanone	1260	1263	MS + LRI	172.65 ± 8.79 ^a^	168.25 ± 9.54 ^a^	165.13 ± 8.40 ^a^	83.94 ± 8.48 ^b^	70.36 ± 7.41 ^c^
4-Pentenoic acid ethyl ester	-	1403	MS	ND	ND	100.72 ± 9.11	65.90 ± 5.47	ND
1-Decene	989	992	MS + LRI	ND	ND	197.02 ± 8.48 ^c^	235.26 ± 8.89 ^b^	413.73 ± 13.09 ^a^
Undecane	1100	1103	MS + LRI	34.87 ± 5.83 ^a^	60.45 ± 4.83 ^a^	67.84 ± 6.49 ^a^	73.75 ± 9.50 ^a^	130.01 ± 15.24 ^a^
Dodecane	1200	1204	MS + LRI	68.89 ± 9.28 ^c^	98.82 ± 5.93 ^b^	207.11 ± 5.40 ^a^	ND	ND
Tridecane	1300	1301	MS + LRI	11.49 ± 2.48 ^e^	36.25 ± 2.36 ^d^	66.96 ± 5.96 ^c^	87.88 ± 4.47 ^b^	127.14 ± 12.63 ^a^
3-Methyltridecane	1371	1373	MS + LRI	ND	82.60 ± 67.70	ND	ND	ND
2,6,10-Trimethyldodecane	1376	1380	MS + LRI	ND	81.45 ± 11.60 ^c^	252.42 ± 11.91 ^a^	207.31 ± 5.71 ^b^	51.90 ± 5.44 ^d^
1-Tetradecene	1396	1400	MS + LRI	ND	ND	337.85 ± 19.89 ^b^	356.20 ± 8.21 ^b^	437.06 ± 28.48 ^a^
Tetradecane	1400	1402	MS + LRI	9.95 ± 0.43 ^d^	68.02 ± 9.59 ^c^	99.89 ± 8.50 ^a^	85.76 ± 16.42 ^b^	70.99 ± 6.60 ^c^
Hexadecane	1600	1605	MS + LRI	4.57 ± 1.41 ^c^	7.93 ± 2.60 ^c^	31.56 ± 9.36 ^b^	39.22 ± 55.01 ^ab^	43.64 ± 6.50 ^a^
Nonadecane	1900	1901	MS + LRI	ND	ND	121.53 ± 8.23^a^	84.57 ± 43.95 ^b^	24.13 ± 4.88 ^c^
Oxirane, 2-octyl-	-	1264	MS + LRI	ND	ND	ND	ND	93.15 ± 7.76
2,7-Dimethyloctane	-	1172	MS + LRI	ND	ND	ND	ND	32.03 ± 7.38
( ± )-Limonene	-	1081	MS + LRI	307.89 ± 7.53 ^e^	398.55 ± 9.33 ^d^	540.20 ± 17.41 ^c^	687.57 ± 13.56 ^b^	917.28 ± 9.11 ^a^
3,5-Dimethyl-1-Hexene	-	1639	MS + LRI	ND	ND	ND	ND	248.17 ± 12.26
Toluene	757	760	MS + LRI	127.40 ± 9.75 ^b^	129.56 ± 7.83 ^b^	244.88 ± 12.00 ^a^	257.23 ± 6.11 ^a^	102.35 ± 12.96 ^c^
2,5-Dimethylpyrazine	917	920	MS + LRI	ND	ND	ND	ND	586.28 ± 15.37
2-Amylfuran	991	993	MS + LRI	47.87 ± 7.82	ND	ND	ND	ND
cis-Anethol	1286	1290	MS + LRI	42.93 ± 9.78 ^d^	45.90 ± 4.09 ^cd^	56.61 ± 6.05 ^c^	84.66 ± 8.96 ^b^	127.83 ± 9.02 ^a^
2-Ethyl-3,5-dimethylpyrazine	-	1302	MS + LRI	243.59 ± 30.29 ^a^	120.11 ± 3.99 ^b^	87.87 ± 9.82 ^c^	70.11 ± 3.92 ^d^	60.11 ± 3.92 ^e^
2-butyl tetrahydrofuran	-	1325	MS + LRI	155.36 ± 11.52 ^a^	150.36 ± 8.27 ^a^	140.46 ± 10.25 ^a^	74.71 ± 6.68 ^b^	57.96 ± 7.34 ^b^
2-Pentylpyridine	-	1453	MS + LRI	ND	ND	ND	ND	32.32 ± 9.76
2,5-Dimethyltetrahydrofuran	-	1520	MS + LRI	32.54 ± 4.32	ND	ND	ND	ND

Different lowercase letters in the same row indicate that there is significant difference (*p* < 0.05). * The Linear retention index of volatile compounds on HP-5 MS columns and calculated data was based on n-alkanes (C_7_–C_40_). **^+^** Means of identification: MS, mass spectrum comparison using NIST libraries; LRI, linear retention index compared with literature values. ND: volatile compounds not detected. “-”: not reported in the literature. F_0_L_4_ represented the mutton shashliks with 4 lean cubes; F_1_L_3_ represented the mutton shashliks with 1 fat cube and 3 lean cubes; F_2_L_2_ represented the mutton shashliks with 2 fats cubes and 2 lean cubes; F_3_L_1_ represented the mutton shashliks with 3 fat cubes and 1 lean cube; F_4_L_0_ represented the mutton shashliks with 4 fat cubes.

**Table 3:** Volatile compounds in oral processing of roasted mutton shashliks with different fat–lean ratios (μg/kg).

Volatile Compounds	LRI *		Identification ^+^	F_0_L_4_	F_1_L_3_	F_2_L_2_	F_3_L_1_
	Literature	Calculated					
Hexanal	800	802	MS + LRI	91.32 ± 10.97 ^c^	237.98 ± 12.33 ^b^	378.81 ± 10.00 ^a^	397.18 ± 11.26 ^a^
Heptanal	901	904	MS + LRI	69.14 ± 5.23 ^c^	232.18 ± 2.17 ^b^	351.09 ± 16.74 ^a^	374.97 ± 12.63 ^a^
Octanal	1004	1005	MS + LRI	63.13 ± 2.42 ^c^	160.22 ± 7.29 ^b^	264.52 ± 12.96 ^a^	280.03 ± 18.29 ^a^
Nonanal	1112	1114	MS + LRI	94.62 ± 10.03 ^c^	181.54 ± 11.6 ^b^	206.70 ± 16.03 ^a^	213.49 ± 12.64 ^a^
Decanal	1202	1205	MS + LRI	121.43 ± 6.47 ^c^	123.90 ± 9.11 ^c^	143.39 ± 9.55 ^b^	277.83 ± 11.08 ^a^
(2E)-2-Decenal	1265	1268	MS + LRI	ND	29.04 ± 1.86 ^b^	50.34 ± 2.03 ^a^	54.06 ± 1.98 ^a^
7-Hydroxy-3,7-dimethyloctanal	1300	1303	MS + LRI	ND	ND	ND	118.10 ± 4.03 ^a^
Ethyl hexanoate	998	1000	MS + LRI	ND	63.49 ± 2.65 ^c^	103.54 ± 1.61 ^a^	94.32 ± 2.31 ^b^
γ-Caprolactone	1055	1058	MS + LRI	ND	ND	74.15 ± 4.01 ^a^	57.32 ± 1.51 ^b^
(±)-Limonene	-	1081	MS	75.53 ± 3.75 ^c^	183.83 ± 6.24 ^b^	205.24 ± 6.98 ^a^	207.29 ± 6.01 ^a^
Undecane	1100	1103	MS + LRI	ND	66.67 ± 2.36 ^b^	80.33 ± 1.87 ^a^	82.14 ± 3.35 ^a^
Dodecane	1200	1204	MS + LRI	ND	60.77 ± 4.36	ND	ND
2,6,10-Trimethyldodecane	1376	1380	MS + LRI	ND	ND	48.34 ± 3.02 ^b^	63.75 ± 0.80 ^a^
1-Tetradecene	1396	1400	MS + LRI	ND	ND	41.29 ± 1.12	ND
Tetradecane	1400	1402	MS + LRI	ND	ND	61.58 ± 4.43 ^a^	49.34 ± 4.54 ^b^
Hexadecane	1600	1605	MS + LRI	ND	46.42 ± 2.15 ^c^	74.02 ± 6.26 ^b^	97.00 ± 0.02 ^a^
2,5-Dimethylpyrazine	917	920	MS + LRI	32.98 ± 0.18 ^b^	ND	ND	118.10 ± 4.03 ^a^
1-Heptanol	969	973	MS + LRI	ND	ND	33.40 ± 2.55	36.15 ± 3.11
P-Xylene	875	880	MS + LRI	ND	ND	112.55 ± 36.38	ND
cis-Anethol	1286	1290	MS + LRI	62.17 ± 2.32 ^c^	141.35 ± 5.33 ^b^	201.82 ± 12.44 ^a^	196.00 ± 8.93 ^a^
2-Ethyl-3,5-dimethylpyrazine	-	1302	MS + LRI	33.69 ± 2.31 ^c^	93.15 ± 5.75 ^ab^	87.93 ± 2.81 ^b^	97.29 ± 4.88 ^a^

Different lowercase letters in the same row indicate that there is significant difference (*p* < 0.05). * The Linear retention index of volatile compounds on HP-5 MS columns and calculated data was based on n-alkanes (C_7_–C_40_). **^+^** Means of identification: MS, mass spectrum comparison using NIST libraries; LRI, linear retention index compared with literature values. ND: volatile compounds not detected. “-”: not reported in the literature. F_0_L_4_ represented the mutton shashliks with 4 lean cubes; F_1_L_3_ represented the mutton shashliks with 1 fat cube and 3 lean cubes; F_2_L_2_ represented the mutton shashliks with 2 fats cubes and 2 lean cubes; F_3_L_1_ represented the mutton shashliks with 3 fat cubes and 1 lean cube.

## Data Availability

All related data and methods are presented in this paper. Additional inquiries should be addressed to the corresponding author.

## Supplementary Materials

The following supporting information can be downloaded at: https://www.mdpi.com/article/10.3390/foods12101929/s1, Table S1: Ten electronic nose sensors and their compound detection sensitivity.
