# Supplementary material for: Effect of Fat to Lean Meat Ratios on the Formation of Volatile Compounds in Mutton Shashliks
Source: Foods. 2023 May 9;12(10):1929. doi: 10.3390/foods12101929 (PMC10216914; doi:10.3390/foods12101929)
Supplement: Supplementary file 1 [file foods-12-01929-s001.zip › foods-2366718-supplementary.pdf]

Table S1. Ten electronic nose sensors and their compound detection sensitivity.

Sensor array	Sensor code	Substances for sensing	Sensitivity
R1	W1C	Generally aromatic compounds	Toluene，10 mL/m³
R2	W5S	Highly sensitive to nitrogen oxides	nitrogen dioxide，1 mL/m³
R3	W3C	Ammonia, also sensitive to aromatic compounds	Benzene，10 mL/m³
R4	W6S	High selectivity for hydrogen compounds	hydrogen，100 mL/m³
R5	W5C	Sensitive to short chain alkanes	Propane，1 mL/m³
R6	W1S	Sensitive to methyl compounds	methane，100 mL/m³
R7	W1W	Sensitive to inorganic sulphur compounds	hydrogen sulfide，1 mL/m³
R8	W2S	Sensitive to alcohols and carbonyls (aldehydes and ketones)	carbon monoxide，100 mL/m³
R9	W2W	Reactive with aromatic and organic sulphur compounds	hydrogen sulfide，1 mL/m³
R10	W3S	Selective for long chain alkanes	methane，10 mL/m³
